# Association Study between Novel CYP26 Polymorphisms and the Risk of Betel Quid-Related Malignant Oral Disorders

**DOI:** 10.1155/2015/160185

**Published:** 2015-03-09

**Authors:** Shyh-Jong Wu, Yun-Ju Chen, Tien-Yu Shieh, Chun-Ming Chen, Yen-Yun Wang, Kun-Tsung Lee, Yueh-Ming Lin, Pei-Hsuan Chien, Ping-Ho Chen

**Affiliations:** ^1^Department of Medical Laboratory Science and Biotechnology, Kaohsiung Medical University, No. 100, Shih-Chuan 1st Road, Kaohsiung 80708, Taiwan; ^2^Department of Biological Science & Technology, I-Shou University, No. 1, Section 1, Syuecheng Road, Kaohsiung 84001, Taiwan; ^3^Department of Medical Research, E-Da Hospital, No. 1, Yida Road, Kaohsiung 82445, Taiwan; ^4^School of Oral Hygiene, Taipei Medical University, No. 250, Wuxing Street, Taipei 11031, Taiwan; ^5^School of Dentistry, College of Dental Medicine, Kaohsiung Medical University, No. 100, Shih-Chuan 1st Road, Kaohsiung 807, Taiwan; ^6^Department of Oral and Maxillofacial Surgery, Kaohsiung Medical University Hospital, Kaohsiung Medical University, No. 100, Shih-Chuan 1st Road, Kaohsiung 80708, Taiwan; ^7^Department of Clinical Research, Kaohsiung Medical University Hospital, No. 100, Shih-Chuan 1st Road, Kaohsiung 80708, Taiwan; ^8^Department of Oral Hygiene, College of Dental Medicine, Kaohsiung Medical University, No. 100, Shih-Chuan 1st Road, Kaohsiung 80708, Taiwan; ^9^Division of Colorectal Surgery, Department of Surgery, Kaohsiung Chang Gung Memorial Hospital and Chang Gung University College of Medicine, No. 123, Dapi Road, Kaohsiung 83301, Taiwan

## Abstract

BQ chewing may produce significant amounts of reactive oxygen species (ROS), resulting in oral mucosa damage, and ROS may be metabolized by CYP26 families. Because the CYP26 polymorphisms associated with malignant oral disorders are not well known, we conducted an association study on the associations between the single nucleotide polymorphisms (SNP) of CYP26 families and the risks of malignant oral disorders. BQ chewers with the CYP26A1 rs4411227 C/C+C/G genotype and C allele showed an increased risk of oral and pharyngeal cancer (adjusted odds ratio (aOR) = 2.30 and 1.93, respectively). The CYP26B1 rs3768647 G allele may be associated with oral and pharyngeal cancer (aOR = 3.12) and OPMDs (aOR = 2.23). Subjects with the rs9309462 CT genotype and C allele had an increased risk of oral and pharyngeal cancer (aOR = 9.24 and 8.86, respectively) and OPMDs (aOR = 8.17 and 7.87, respectively). The analysis of joint effects between the CYP26A1 rs4411227 and CYP26B1 rs3768647/rs9309462 polymorphisms revealed statistical significance (aOR = 29.91 and 10.03, respectively). Additionally, we observed a significant mRNA expression of CY26A1 and CYP26B1 in cancerous tissues compared with adjacent noncancerous tissues. Our findings suggest that novel CYP26 polymorphisms are associated with an increased risk of malignant oral disorders, particularly among BQ chewers.

## 1. Introduction

Approximately 600 million people chew betel quid (BQ) in the world [1], primarily in South/Southeast Asia and the South Pacific islands [2]. BQ without tobacco is an addictive and psychostimulant substance and is a group I human carcinogen, as stated in an evaluation by the International Agency for Research on Cancer (IARC) [3, 4]. Additionally, areca nut (AN) is the primary component in BQ, which has also been categorized as a group I human carcinogen by the IARC [4]. BQ usage is increasingly recognized for its association with malignant oral disorders [4–10]. The malignant oral disorders include oral potentially malignant disorders (OPMDs) (i.e., oral submucous fibrosis (OSF), leukoplakia, erythroplakia, and lichen planus) and cancers of the oral cavity and pharynx. Epidemiological studies have indicated that BQ chewing can elevate the risk of malignant oral diseases [4–10]. A recent study found that the percentage of male BQ chewers was more than 85% among oral cancer patients [9].

A previous study suggested that chewing BQ may produce significant reactive oxygen species (ROS), such as the hydroxyl radical, which may induce the oxidative damage of oral tissue [11]. ROS are capable of inducing nucleotide modification and the generation of DNA double stranded breaks [12] and cellular 8-hydroxy-2′-deoxyguanosine (8-OH-dG) induced DNA oxidative damage [13]. In granulocyte-differentiated HL60 cells, a previous report indicated that all-trans retinoid acid (at-RA) induces NADPH oxidase-mediated ROS generation [14].

The cytochrome P450 (CYP) 26 family via oxidative metabolism to partially regulate intracellular RA compounds (such as the concentration of at-RA) affected the balance of retinoic acid (RA) in homeostasis as well as their related signal transduction [15]. RA is a vitamin A-activated metabolite that primarily regulates cell growth, differentiation, and apoptosis in the important mechanism of fetal development as well as adult life activities [16]. RA exhibits its cardioprotective effects by preventing cardiomyocyte apoptosis and ROS generation [17]. The at-RA can produce apoptosis [18] by inducing ROS formation in rat Sertoli cells [18, 19]. In granulocyte-differentiated HL60 cells, at-RA produces NADPH oxidase-mediated ROS formation [14].

In this CYP26 family, there are two major isoforms, CYP26A1 and CYP26B1, that can be induced by RA [15]. RA compounds usually control their content through a precise balancing mechanism. We speculated that BQ use would change RA metabolism via the stimulation of CYP26B1 in the oral mucosa, and the metabolism of RA is crucial for the occurrence of oral cancer [9]. This* in vivo* regulation was primarily through CYP26A1 and CYP26B1 metabolism [20], and we speculated that this regulation may be associated with ROS. Thus, the specific aim of this association study was to investigate the role of CYP26 family (CYP26A1 and CYP26B1) single nucleotide polymorphisms (SNPs) in the risk of OPMDs and oral and pharyngeal cancers.

## 2. Methods

### 2.1. Participants and Data Collection

Patients with oral/pharyngeal cancers (*N* = 211) and OPMDs (*N* = 218) were identified from the Department of Oral and Maxillofacial Surgery, Kaohsiung Medical University Hospital in Taiwan, between 2006 and 2010. Healthy controls (*N* = 218) were recruited from a community oral health survey. All volunteers signed written informed consent and provided whole blood. The study was approved by the Institutional Review Board of Kaohsiung Medical University Hospital (KMUH-IRB-970413, KMUH-IRB-950315, and KMUH-IRB-950094). The characteristics of the demographic variable and the status of substance use (such as alcohol, BQ, and cigarette use) were investigated by trained interviewers. Alcohol drinkers included current and former drinkers. Smokers included current and former smokers. Alcohol users, BQ chewers, and cigarette smokers were defined as alcoholic beverage consumption (irrespective of quantity) at least once per week for longer than 6 months, at least one quid of BQ chewed per day for longer than 6 months, and at least 10 cigarettes smoked per week for longer than 6 months, respectively. A cumulative lifetime BQ exposure (pack-years) was defined as the number of packs consumed multiplied by chewing years. One pack was defined as chewing 10 quids per day. Eight subjects with oral cancerous tissue and adjacent noncancerous oral tissue were collected during necessary surgery resection. These tissue specimens without chemotherapy or radiation therapy were analyzed. Informed consents was also signed by eight oral and pharyngeal patients.

### 2.2. DNA Extraction and Genotyping

Eight c.c. of peripheral blood was collected in tubes containing ethylenediaminetetraacetic acid (EDTA). Genomic DNA was extracted from the peripheral blood samples using the QIAamp DNA Mini Kit (Qiagen) according to the manufacturer's instructions. The extracted DNA samples were stored at −80°C until examination. DNA concentrations were checked via optical density at 260–280 nm (Nano Drop ND-2000; Thermo Fisher Scientific Inc.).

Single-nucleotide polymorphisms (SNPs) of CYP26A1 and CYP26B1 were selected with minor allele frequency from a public reference database in the Chinese HapMap-CHB. SNP Genotyping was performed using a Taqman Genotyping Assay according to the manufacturer's instructions. All assays and gDNA were conducted in 384-well plates, and PCR was performed. After PCR amplification, an endpoint plate read was performed using an Applied Biosystems ViiA7 Real-Time PCR System. The Sequence Detection System (SDS) Software analyzed the fluorescence measurements made during the plate read to plot fluorescence (Rn) values based on the signals from each well. The plotted fluorescence signals indicate which alleles are in each sample.

### 2.3. Real-Time qRT-PCR Analysis

The total RNA was extracted from oral cancerous tissues, as well as their adjacent noncancerous tissue using TRIzol (Invitrogen, Carlsbad, CA, USA) and the commercial protocol of the manufacturer as described [21]. Before further real-time qRT-PCR PCR analysis, each cDNA pool was prepared at −20°C. For real-time PCR assays, specific oligonucleotide primer pairs were purchased from Roche Universal ProbeLibrary. The reactions of real-time qRT-PCR were analyzed using the Roche LightCycler Instrument 1.5 with a LightCycler FastStart DNA Master^PLUS^ SYBR Green I kit (Roche Cat. 03 515 885 001, Castle Hill, Australia). The fold change of the expression of the target gene relative to the internal control gene GAPDH in each sample was calculated using the following formula: 2^−ΔΔCt^  where  ΔCt = Ct_target  gene_ − Ct_internal  control_ and ΔΔCt = ΔCt_test  sample_ − ΔCt_control  sample_.

### 2.4. Statistical Analysis

The control genotype distribution complied with the Hardy-Weinberg equilibrium (*P* ≥ 0.05). In this statistical analysis, the questionnaire data included the demographic information, substance use (alcohol, betel quid, and cigarette use), and history and disease status (normal controls, OPMDs, and oral/pharynx cancer). General linear model (GLM) analysis was used for comparing the differences in the means between three groups, and post hoc comparisons were analyzed using the Bonferroni test. Using a multinomial logistic regression model to control for potential confounders, such as demographic factors (continuous age, ethnicity, and education levels) and substance use (cigarette and alcohol use), an exact *P* value, adjusted odds ratio (aOR), and 95% confidence interval (CI) were produced for our tables. All statistical analyses were performed using the SAS Statistical Package (Version 9.1.3, SAS Institute Inc.).

## 3. Results

All subjects (*N* = 485) were BQ chewers. Among these, 56 OPMDs patients, 211 oral and pharyngeal cancer patients, and 218 healthy controls were recruited in this case-control study. The demographic characteristics, alcohol use status, and cigarette use status are shown in Table 1. There was statistical significance in the average ages among oral and pharyngeal cancer patients, OPMDs patients, and healthy controls (50.12 ± 9.15, 49.19 ± 11.18, and 43.57 ± 8.58 years old, respectively). The distribution of ethnicity, education levels, alcohol drinking status, and cigarette smoking status showed no statistically significant differences. Oral and pharyngeal cancer patients exhibited significantly older age at drinking initiation compared with controls (*P* < 0.05). The oral and pharyngeal cancer and OPMDs patients showed a significantly higher average amount of smoking than the controls (*P* < 0.05). In terms of BQ chewing, we observed that oral and pharyngeal cancer and OPMDs patients had a significantly longer duration of chewing and older age at chewing initiation than the controls (*P* < 0.05). Additionally, oral and pharyngeal cancer and OPMDs patients had higher cumulative lifetime BQ use compared with the controls.

### 3.1. The Distribution of Genetic Polymorphisms between Oral and Pharyngeal Patients and Control Groups

Between the oral and pharyngeal patients and control groups, the difference in genotype frequency distribution was statistically significant for CYP26A1 rs4411227 (Table 2). After adjusting for covariates (age, ethnicity, education, alcohol drinking, and cigarette smoking), the results showed that BQ chewers with the rs4411227 C/G genotype or C/C+C/G combined genotype had an approximately 2-fold increased risk for the development of oral and pharyngeal cancer relative to those with the G/G genotype. The subjects with the C allele had a significantly higher risk of oral and pharyngeal cancer than those in the control groups (aOR = 1.93; 95% CI = 1.30–2.88). There were no significant differences in the genotype distribution of CYP26B1 rs887844 between the oral and pharyngeal patients and the controls (*P* values >0.05). Compared with the control groups, the subjects with the rs3768647 G allele had a significantly higher independent risk for oral and pharyngeal cancer (aOR = 3.12; 95%  CI = 2.28–4.27). After adjusting for covariates, the subjects with the rs9309462 C/T genotype (aOR = 9.24; 95% CI = 1.90–45.00) or C allele (aOR = 8.86; 95%  CI = 1.84–42.59) had a significantly higher independent risk for oral and pharyngeal cancer compared with the control groups.

### 3.2. The Distribution of Genetic Polymorphisms between the OPMDs Patients and Control Groups

There were no differences in rs4411227 polymorphism distribution between the OPMDs patients and controls (Table 2). After adjusting the covariates (age, ethnicity, education, alcohol drinking, and cigarette smoking), the subjects carrying the rs887844 A/G genotype had a marginally enhanced risk for OPMDs (aOR = 1.87; 95%  CI = 1.01–3.49; *P* = 0.0482) compared with the G/G type. Individuals with the rs3768647 G allele had a 2.23-fold greater risk of OPMDs compared with those with the C allele (aOR = 2.23; 95%  CI = 1.40–3.54). The subjects with the rs9309462 C/T genotype and C allele showed a significantly higher risk compared with the subjects with the TT and T allele genotype (aOR = 8.17; 7.87, respectively).

### 3.3. The Gene-Gene Joint Effects in the Risk of Oral and Pharyngeal Cancer and OPMDs

We further analyzed the gene-gene joint effects in the risk of oral and pharyngeal cancer and OPMDs (Table 3), after adjusting the covariates (age, ethnicity, education, alcohol drinking, and cigarette smoking). BQ chewers with the CYP26A1 rs4411227 C allele and CYP26B1 rs3768647 G allele had the highest risk of oral and pharyngeal cancer compared with the subjects carrying the CYP26A1 rs4411227 G allele and CYP26B1 rs3768647 C allele (aOR = 29.91; 95%  CI = 10.75–83.23). Similarly, individuals carrying the CYP26A1 rs4411227 C allele and CYP26B1 rs3768647 G allele had the highest risk of OPMDs relative to the subjects carrying the CYP26A1 rs4411227 G allele and CYP26B1 rs3768647 C allele (aOR = 11.25; 95%  CI = 3.18–39.77). BQ chewers with the CYP26A1 rs4411227 C allele and CYP26B1 rs9309462 C allele had the highest risk of oral and pharyngeal cancer compared with the subjects carrying the CYP26A1 rs4411227 G allele and CYP26B1 rs9309462 T allele (aOR = 10.03; 95%  CI = 1.05–95.60). However, there were no significant differences on gene-gene joint effects between the presence of the CYP26A1 rs4411227 and CYP26B1 rs9309462 genetic variations regarding the risk of OPMDs.

### 3.4. The CYP26A1 and CYP26B1 mRNA Expression of Oral Paired Tissue and Adjacent Noncancerous Tissues

**Figure 1:**
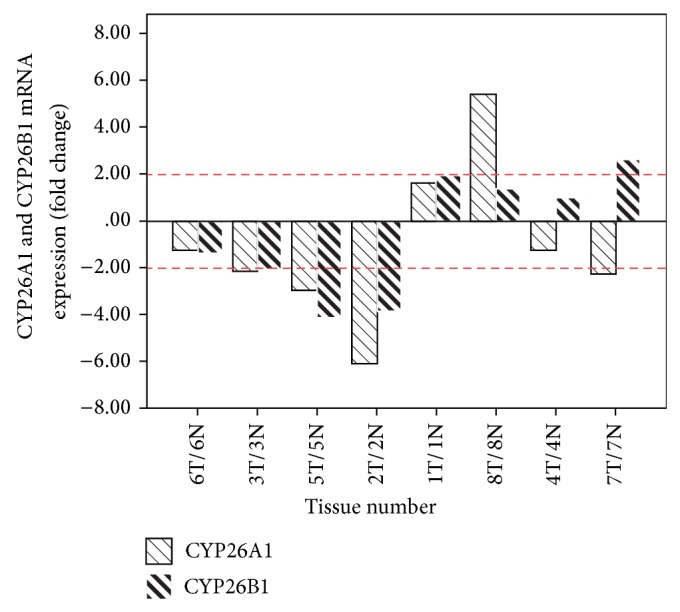
The induced mRNA of CYP26A1 and CYP26B1 in human oral cancer tissue (T) and its adjacent normal tissue (N). Paired tissue samples (tumor and adjacent normal tissue) without chemotherapy/radiation therapy were analyzed. Compared with human adjacent tissue (*N* = 8), the relative fold change was calculated in triplicate (columns, mean; bars, SD) using the formula 2^−ΔΔCt^.

We investigated CYP26A1 and CYP26B1 quantitative mRNA in expression in eight patients (numbers 1, 2, 3, 4, 5, 6, 7, and 8) (Figure 1). Compared with their adjacent noncancerous tissues, tumor tissues exhibited the consistent downregulation mRNA of CYP26A1 and CYP26B1 in patients number 2, 3, 5, and 6 (expression >2-fold change in numbers 2, 3, and 5). In patients number 1 and 8, the upregulation of the expression of CYP26A1 and CYP26B1in cancerous tissue was observed, compared with their adjacent noncancerous tissues. In the cancer tissue of number 4, a slightly decreased expression of CYP26A1 and increased expression of CYP26B1 were found. CYP26A1 expression was lower in the cancer tissue of number 7 compared with its adjacent tissue, but the expression of CYP26B1 was higher in the cancer tissue than in the adjacent tissue.

## 4. Discussion

The International Agency for Research on Cancer (IARC) has indicated that betel quid without tobacco can cause oral cancer and has stated that, in experimental animals, there is sufficient evidence to establish the carcinogenicity of the areca nut; there is limited evidence for the carcinogenicity of arecoline [4]. The areca nut (AN) is a major ingredient of BQ, and arecoline is the most abundant AN alkaloid. In detoxifying AN or arecoline, two monooxygenase systems (cytochrome P450 and flavin-containing monooxygenases) are implicated in phase I metabolism [22]. A previous report indicated that the monooxygenase activity of CYP26B1 may be involved in the detoxification process of BQ chewing [22]. Furthermore, CYP26B1 has been demonstrated to participate in the metabolism of at-RA and has been indicated to play a major role in the protection of specific tissues for at-RA exposure [23]. A recent study demonstrated that the retinoic acid-metabolizing enzymes CYP26A1 and CYP26B1 are significantly overexpressed in colorectal cancer tissue [24].

This is the first study to indicate that the mRNA expression of CYP26 families (CYP26A1 and CYP26B1) and their SNP variants play a novel role in the occurrence or developmental mechanism of malignant oral disorders. In our analysis, we evaluated the risk effect of CYP polymorphisms among BQ chewers. These findings showed that BQ chewers with CYP26A1 risk polymorphism (rs4411227) may enhance the risk of oral and pharyngeal cancer. Also, subjects carrying CYP26B1 risk polymorphism (rs3768647/rs9309462) have an increased susceptibility to oral malignant disorders. The CYP26A1 rs4411227 and CYP26B1 rs3768647/rs9309462 may have significant joint effects in the risk of oral malignant disorders (particularly in oral and pharyngeal patients) among BQ chewers. To rule out differences in gene expression between different individuals, we collected paired oral tissues to observe the significant expression of CYP26A1 and CYP26B1. Overall, these findings seem to have strengths on the role of CYP26A1 and CYP26B1 in the etiology of oral malignant disorders.

### 4.1. The Susceptible Metabolic CYP26B1 Gene

The CYP26B1 gene is located on chromosome 2p13.2 and covers a total of eighteen thousand base pairs. After transcription, the CYP26B1 gene formed 6 exons and 8.57 kb introns and included an approximately 3 kb long untranslated 3^′  ^region [25, 26]. It is also a single-oxygenase enzyme (monooxygenase) that catalyzes many reactions, such as those involving drug metabolism and the synthesis of hormones, cholesterol, and lipids. However, the catalytic function of CYP26B1 can catalyze at-RA into a hydroxylated form; this also refers to the process of the oxidation of RA through the added oxygen in number 4 seat of the carbon skeleton, subsequently metabolizing RA into the polar and inactive form (such as 4-oxo-,4-OH-,5,6-epoxy and 18-OH-all-trans-retinoic acid) to activate it [27] while not affecting the cell physiology. A previous report suggested that CYP26B1 appears to be necessary in the physiological role of RA catabolism, whereas CYP26A1 played an important role given excessive RA in the cells [28].

### 4.2. The Susceptible Metabolic CYP26A1 Gene

CYP26A1 is located on chromosome 10q23-q24 [29]. CYP26A1 and CYP26B1 are at-RA hydroxylases that are responsible for the catalytic formation of similar metabolites in a cellular system; there is only 40% similarity among the CYP26A1 gene sequence and CYP26B1 gene sequence [30]. CYP26A1 is a hydroxylation enzyme for the major metabolism of RA and transforms RA into an inactive RA hydroxy derivative [31, 32]. CYP26A1 has high specificity for at-RA and oxidation RA to form 4-OH-RA, 18-OH-RA, and 4-oxo-RA [33]. Scholars found that CYP26A1 had a higher catalytic ability compared with CYP26B1 and that CYP26A1 was primarily responsible for the metabolism of at-RA and provides a protective barrier to avoid at-RA overexposure [30]. CYP26A1 may be associated with the metabolism of RA in human epidermal keratinocytes [28]. A previous report indicated that, in long term sunlight-damaged skin cells and in the increased expression of RA-metabolizing enzymes, CYP26A1 may cause a deficiency of vitamin A, which could potentially lead to the malignant transformation of keratinocytes in the early development of skin cancer [34].

A review article indicated that the inhibition of CYP26A1 expression reduces tumorigenicity through the use of RA metabolism blocking agents (RAMBAs) [15]. Previous studies demonstrated that an increased expression of CYP26A1 was found in human familial adenomatous polyposis adenomas, sporadic colon cancers, and primary ovarian cancer [20, 35]. A report noted that RA can induce CYP26A1 expression in neuroblastoma, breast cancer, and lung cancer cell lines [36]. In breast cancer or colon cancer cells, CYP26A1 gene expression can be induced via the receptor of vitamin A [37]. In breast epithelial adenocarcinoma tissue cultures, head and neck squamous cell carcinoma cells, and acute promyelocytic leukemia (acute promyelocytic leukemia) cells, an increased expression of CYP26A1 and increased catabolic activity of RA can be detected [38–40].

Additionally, 42% (27/65) of tissue samples removed from breast cancer patients had CYP26A1 overexpression; CYP26A1 overexpression may induce intracellular RA consumption, thus pushing the cells toward tumorigenicity; CYP26A1 may be recommended as a candidate oncogene [41]. Researchers found that some CYP450 genes (e.g., CYP26A1) in primary ovarian cancer have significantly higher expressions compared with normal ovarian tissues [35]. Additionally, CYP26A1 overexpression in Barrett's esophagus adenocarcinoma may cause the consumption of intracellular vitamin A acid [42], whereas other reports found that the expression of CYP26A1 is lower in normal human epidermal cells [43, 44]. After RA treatment, at-RA turnover rates are approximately 18-fold higher in squamous head and neck cancer cell lines compared with normal oral keratinocytes; 4-oxo-RA and 4-hydroxy-RA are also generated in the former. Two squamous head and neck cancer cell lines have increased expressions of CYP26 A1 mRNA and showed the highest metabolism of RA [33]. In head and neck cancer patients, the adjacent normal oral keratinocytes showed a 15-fold higher normal oral keratinocyte turnover rate compared with noncancer patients [45]. The above results suggest that RA metabolism potentially played a role in the development of oral cancer. Similarly, recent studies indicated that the increased expression of CYP26A1 genes was related to head and neck cancer [33]. This study also suggested that increasing concentrations of endogenous RA and CYP26A1 inhibitors will be applied in the future treatment of cancer or novel therapies for skin diseases [15, 46].

### 4.3. Retinoic Acid (RA) and Cancer Development

Previous studies reported that the damage of normal RA homeostasis signaling was associated with the development of cancer [47]. The damage to normal RA signaling may be due to the decreased expression of the RA receptor, the decreased transcriptional response of the RA target gene, and increased RA metabolism [48]. Since 1920, vitamin A deficiency has been associated with cell carcinogenesis [49]. Vitamin A deficiency may be associated with increased susceptibility to cancer, and low amounts of vitamin A intake may increase the risk of human cancer [50].

Research data indicated that increased RA compound intake can reduce different varieties of squamous cell carcinoma (such as oral cancer, lung cancer, pharynx cancer, cervical cancer, and bladder cancer); therefore, the deficiency of RA ingestion may result in excessive cell proliferation (hyperplasia) and hyperkeratosis and cause carcinogenesis in oral cavity cells [51]. RA compounds and their isomers can be applied to treat or prevent cancer and skin diseases [48]. A series of intervention studies has indicated that vitamin A can effectively reduce the remission of betel chewers with oral leukoplakia and that it suppressed the occurrence of new oral lesions [52–54]. Previous studies recommended the use of RA treatment for the remission of malignant transformation among BQ chewers with OPMDs; this mechanism may be due to the inhibition of BQ compounds promoting carcinogenesis rather than to them inhibiting the initiation of carcinogenesis development [52, 54, 55]. Additionally, a review article suggested that the inhibition of CYP26 enzyme activity could help to increase the half-life of RA and would be clinically effective for future applications.

CYP polymorphisms related ROS provide important insight into the importance of clinical diagnostic tools (e.g., screen test of SNP) in BQ chewers for the prevention of malignant oral disorders [56]. The study limitations were a smaller sample size for evaluation of mRNA expression and lack of phenotype information among patients. In the future research, we will collect sufficient samples to confirm expression of CYP26A1 and CYP26 B1 in betel quid-related malignant oral disorders.

In conclusion, this study suggested that BQ chewers with ROS related to CYP26A1 and CYP26B1 polymorphisms are associated with an increased risk of oral and pharyngeal cancer and OPMDs. Our findings may be useful in identifying subjects who are at an increased risk for the development of oral malignant disorders.

## Figures and Tables

**Table 1:** Distribution of male betel quid chewers associated with characteristics of selected demographic factors.

	Oral and pharyngeal cancer (*N* = 211)	OPMDs (*N* = 56)	Control (*N* = 218)	*P* value
BQ chewers	*N* (%)^a^	*N* (%)	*N* (%)	
Age (mean ± S.D., years)	50.12 ± 9.15	49.19 ± 11.18	43.57 ± 8.58	<0.05
Ethnicity				
Hokkien	181 (85.78)	44 (78.57)	174 (79.82)	0.20
Others	30 (14.22)	12 (21.43)	44 (20.18)	
Education (years)				
≤9	127 (60.19)	29 (51.79)	141 (64.68)	0.19
>9	84 (39.81)	27 (48.21)	77 (35.32)	
Alcohol drinking status				
Nondrinkers	68 (31.28)	16 (28.57)	55 (25.23)	0.38
Drinkers	145 (68.72)	40 (71.43)	163 (74.77)	
Age at starting drinking (mean ± S.D., years)	22.58 ± 6.89	20.14 ± 4.85	18.60 ± 4.73	<0.05
Years of alcohol drinking	24.32 ± 8.63	26.94 ± 10.23	19.21 ± 8.29	<0.05
Cigarette smoking status				
Nonsmokers	16 (7.58)	4 (7.14)	8 (3.67)	0.20
Smokers	195 (92.42)	52 (92.86)	210 (96.33)	
Age at starting smoking (mean ± S.D., years)	19.01 ± 4.06	19.35 ± 5.08	17.38 ± 9.77	0.05
Average amount of smoking (cigarette/day)	26.12 ± 14.47	27.10 ± 15.18	16.06 ± 11.03	<0.05
Years of cigarette smoking	27.89 ± 9.20	28.96 ± 9.12	26.19 ± 7.22	0.05
BQ chewing status				
Age at starting chewing (mean ± S.D., years)	22.17 ± 6.53	22.66 ± 8.23	18.99 ± 5.07	<0.05
Years of BQ chewing	21.96 ± 8.55	21.39 ± 9.44	18.60 ± 9.16	<0.05
Average amount of chewing (quids/day)	34.01 ± 35.39	34.66 ± 27.10	30.83 ± 34.99	0.59
Cumulative lifetime BQ use (pack-years)^a^	73.91 ± 73.58	69.78 ± 53.28	58.73 ± 72.09	0.10

The *P* < 0.05 indicated statistical significance, and it was calculated via the Chi-square or GLM test (post hoc was compared using the Bonferroni test). Means within each row (in capital letter) followed by the different letter are statistically significant differences (via the Bonferroni test (*P* < 0.05)).

^a^One chewed pack corresponds to 10 betel quids.

**Table 2:** Distribution of CYP26 families genotype and allele frequency among malignant oral disorders patients and control groups.

	Oral and pharyngeal cancer (*N* = 211)	OPMDs (*N* = 56)	Controls (*N* = 218)	Oral and pharyngeal cancer versus controls		OPMDs versus controls	
BQ chewers	*N* (%)	*N* (%)	*N* (%)	aOR (95% CI)	*P*	aOR (95% CI)	*P*
CYP26A1							
rs4411227							
Genotype							
G/G	130 (61.61)	40 (71.43)	170 (77.98)	1.00		1.00	
C/G	74 (35.07)	14 (25.00)	43 (19.72)	2.38 (1.48–3.84)^b∗^	0.0004	1.39 (0.67–2.90)	0.3792
C/C	7 (3.32)	2 (3.57)	5 (2.29)	1.65 (0.46–5.95)	0.4417	0.80 (0.09–7.46)	0.8430
Combined genotype							
G/G	130 (61.61)	40 (71.43)	170 (77.98)	1.00		1.00	
C/C + C/G	81 (38.39)	16 (28.57)	48 (22.02)	2.30 (1.45–3.64)^*^	0.0004	1.33 (0.65–2.70)	0.4389
Allele							
G	334 (79.15)	94 (83.93)	383 (87.84)	1.00		1.00	
C	88 (20.85)	18 (16.07)	53 (12.16)	1.93 (1.30–2.88)^*^	0.0012	1.22 (0.65–2.29)	0.5447
CYP26B1							
rs887844							
Genotype							
G/G	115 (54.50)	25 (44.64)	133 (61.01)	1.00		1.00	
A/G	96 (45.50)	31 (55.36)	85 (38.99)	1.38 (0.91–2.09)	0.1273	1.87 (1.01–3.49)^*^	0.0482
Allele							
G	326 (77.25)	81 (72.32)	351 (80.50)	1.00		1.00	
A	96 (22.75)	31 (27.68)	85 (19.50)	1.26 (0.89–1.80)	0.1941	1.55 (0.93–2.57)	0.0898
rs3768647							
C/G	103 (48.82)	34 (60.71)	218 (100.00)	1.00		1.00	
G/G	108 (51.18)	22 (39.29)	0 (0.00)	—^a^		—^a^	
Allele							
C	103 (24.41)	34 (30.36)	218 (50.00)	1.00		1.00	
G	319 (75.59)	78 (69.64)	218 (50.00)	3.12 (2.28–4.27)^*^	<0.0001	2.23 (1.40–3.54)^*^	0.0007
rs9309462							
T/T	198 (93.84)	52 (92.86)	216 (99.08)	1.00		1.00	
C/T	13 (6.16)	4 (7.14)	2 (0.92)	9.24 (1.90–45.00)^*^	0.0059	8.17 (1.25–53.52)^*^	0.0285
Allele							
T	409 (96.92)	108 (96.43)	434 (99.54)	1.00		1.00	
C	13 (3.08)	4 (3.57)	2 (0.46)	8.86 (1.84–42.59)^*^	0.0065	7.87 (1.22–50.54)^*^	0.0298

aOR was adjusted by continuous age, ethnicity, education level, alcohol drinking, and cigarette smoking habits.

^a^Nonestimated: because the number of samples is equal to zero.

^b∗^ *P* < 0.05.

**Table 3:** Joint effects between CYP26A1 and CYP26B1 polymorphisms among malignant oral disorders patients and control groups.

		Oral and pharyngeal cancer (*N* = 211)	OPMDs (*N* = 56)	Controls (*N* = 218)	Oral and pharyngeal cancer versus controls	OPMDs versus controls
BQ chewers		*N* (%)	*N* (%)	*N* (%)	aOR (95% CI)	aOR (95% CI)
CYP26A1 rs4411227	CYP26B1 rs887844					
Allele	Allele					
G	G	272 (64.45)	75 (66.96)	298 (68.35)	1.00	
C	G	54 (12.80)	6 (5.36)	53 (12.16)	1.13 (0.72–1.76)	0.39 (0.15–1.02)
G	A	62 (14.69)	19 (16.96)	85 (19.50)	0.83 (0.56–1.23)	0.90 (0.50–1.62)
C	A	34 (8.06)	12 (10.71)	0 (0.00)	—^a^	—^a^
CYP26A1 rs4411227	CYP26B1 rs3768647					
Allele	Allele					
G	C	62 (14.69)	26 (23.21)	170 (38.99)	1.00	1.00
C	C	41 (9.72)	8 (7.14)	48 (11.01)	2.51 (1.45–4.34)^b∗^	1.17 (0.48–2.82)
G	G	272 (64.45)	68 (60.71)	213 (48.85)	3.64 (2.51–5.26)^*^	2.14 (1.28–3.59)^*^
C	G	47 (11.14)	10 (8.93)	5 (1.15)	29.91 (10.75–83.23)^*^	11.25 (3.18–39.77)^*^
CYP26A1 rs4411227	CYP26B1 rs9309462					
Allele	Allele					
G	T	327 (77.49)	92 (82.14)	382 (87.61)	1.00	1.00
C	T	82 (19.43)	16 (14.29)	52 (11.93)	1.85 (1.23–2.77)^*^	1.07 (0.55–2.08)
G	C	7 (1.66)	2 (1.79)	1 (0.23)	9.44 (1.08–82.43)^*^	4.58 (0.27–77.85)
C	C	6 (1.42)	2 (1.79)	1 (0.23)	10.03 (1.05–95.60)^*^	12.20 (0.99–151.07)

aOR was adjusted by continuous age, ethnicity, education level, alcohol drinking, and cigarette smoking habits.

^a^Nonestimated: because the number of samples is equal to zero.

^b∗^ *P* < 0.05
